# Fluid Extracts

**Published:** 1868-04-15

**Authors:** 


### *Fluid Extracts.*

The attention of physicians has been turned of late to the general unreliability, and, sometimes, entire worthlessness of the fluid extracts in common use. This inefficiency may be due to the quality of the drug used, or the dishonesty of the manufacturer, but is, in most cases, more probably, the result of the mode of preparation. The trouble seems to lie with those drugs whose medicinal effect depends on volatile principles, which would be evolved on application of even a low degree of heat.

Here, then, is the difficulty that the use of heat renders the extract valueless, because it deprives it of its only valuable ingredient. To dispense with this dangerous agent is the effort of every manufacturer.

Different makers have adopted different methods, involving the use, however, of more or less heat, but none have achieved the result desired, till Dr. Samuel P. Duffield, of Detroit, announced his process, in which he avoids the use of any heat whatever. The following is a short description of this valuable improvement:

"The drug is ground to a coarse powder and placed dry in an iron cylinder. The air is then exhausted by means of an air pump, causing the pores of the drug to give up the air contained in them, and permit the entrance of the menstruum, which is forcibly sucked in through a syphon tube. The effect of this is to impregnate the menstruum with the entire soluble and medicinal properties of the drug, and thus rendering after concentration with the aid of heat unnecessary."

Many of our leading physicians, among whom we might mention Professor Weber and Scott, of Cleveland, Professor Armor, formerly of the Michigan University, Professor Hildreth, of Chicago, have tried fluid extracts made according to Dr. Duffield's process, by Duffield, Parke & Co., of Detroit, with a view to thoroughly test the merits, and have pronounced them decidedly superior to others in use.

In general appearance they differ much from the dark colored preparations to which we are accustomed. The standard is that of the U. S. Pharmacopœia, sixteen troy ounces of the drug to the fluid pint.

